# Robustness in spatially driven bistability in signaling systems

**DOI:** 10.1038/s41598-020-62412-1

**Published:** 2020-03-27

**Authors:** Debora Tenenbaum, Juan Ignacio Marrone, Hernán E. Grecco, Alejandra C. Ventura

**Affiliations:** 1 Departamento de Física, Facultad de Ciencias Exactas y Naturales, Universidad de Buenos Aires, Ciudad Universitaria, Buenos Aires, C1428EHA Argentina; 2 Physics Institute of Buenos Aires, National Research Council (CONICET), Buenos Aires, Argentina; 3 Department of Physiology, Molecular and Cellular Biology, University of Buenos Aires, Buenos Aires, Argentina; 4 Institute of Physiology, Molecular Biology and Neurosciences, National Research Council (CONICET), Buenos Aires, Argentina; 5 Present Address: Department of Physics, Brandeis University, Waltham, MA 02454 and Department of Biochemistry, Brandeis University, Waltham, MA 02454 USA

**Keywords:** Cellular signalling networks, Applied mathematics

## Abstract

Biological systems are spatially organized. This microscopic heterogeneity has been shown to produce emergent complex behaviors such as bistability. Even though the connection between spatiality and dynamic response is essential to understand biological output, its robustness and extent has not been sufficiently explored. This work focuses on a previously described system which is composed of two monostable modules acting on different cellular compartments and sharing species through linear shuttling reactions. One of the two main purposes of this paper is to quantify the frequency of occurrence of bistability throughout the parameter space and to identify which parameters and in which value ranges control the emergence and the properties of bistability. We found that a very small fraction of the sampled parameter space produced a bistable response. Most importantly, shuttling parameters were among the most influential ones to control this property. The other goal of this paper is to simplify the same system as much as possible without losing compartment-induced bistability. This procedure provided a simplified model that still connects two monostable systems by a reduced set of linear shuttling reactions that circulates all the species around the two compartments. Bistable systems are one of the main building blocks of more complex behaviors such as oscillations, memory, and digitalization. Therefore, we expect that the proposed minimal system provides insight into how these behaviors can arise from compartmentalization.

## Introduction

**Figure 1:**
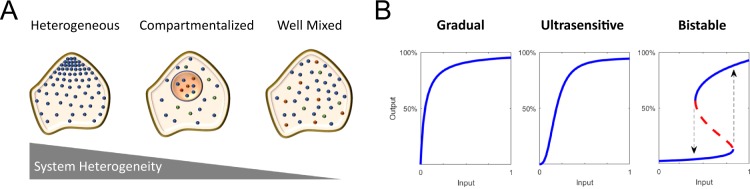
Spatially heterogeneous cellular networks and their function. (**A**) Degrees of spatial heterogeneity. One common approach to dealing with spatial heterogeneities is using physical, chemical and/or biological gradients. A simpler strategy consists in considering heterogeneous systems as composed by different compartments, which can interchange certain molecular species. In the other extreme, heterogeneity is not considered, and a well-mixed system is modeled. (**B**) Examples of different input/output behaviors that a system can exhibit. Hyperbolic responses increase gradually with stimuli, eventually reaching a plateau as the pathway saturates. Sigmoidal responses switch between high and low levels (all-or-none) when the stimulus reaches a threshold level. Bistable responses are discontinuous and exhibit hysteresis.

Eukaryotic cells are spatially organized systems in which different molecules present distinct degrees of heterogeneity (Fig. 1A). Most of the molecules involved in signal transduction systems must shuttle constantly between intracellular compartments to fulfill their specific functions. There are clear benefits to restricting signaling molecules to specific sub-cellular compartments^1^. Keeping them close to their correct targets and regulators, and far from the incorrect ones, may lead to increased specificity in signal transmission^2^. Also, spatially confining signaling molecules increases their local concentration, making encounters between them more frequent, and therefore increasing the velocity of signal transmission. From an information transfer point of view, spatially inhomogeneous systems allow increasing the computational capacity by allowing different biochemical reactions to evolve simultaneously in different parts of the cell^3^.

Aside from these evident benefits, regulated translocation of molecules presents yet another potentially important consequence: it can, in principle, modify the nature of the response of a system allowing enriched dynamical response. In this work we are mainly concerned with bistable responses, which have been shown to be an emergent outcome of a compartmentalized monostable activation/deactivation cycle^4^. Bistable responses, such as the one of p42 MAPK in oocyte maturation^5^, is one of the many input-output relations by which signal processing networks transform simple signals into complex responses, finely tuned for the physiological functions they control. Unlike ultrasensitive or gradual responses (Fig. 1B) intermediate values are impossible as the stimulus-response curve shows an actual discontinuity. Bistable responses exhibit hysteresis: once the stimulus is high enough to turn the system on, it will remain in the *on* state even after the stimulus is lowered below the threshold^6^. Only after the stimulus is decreased below a deactivating threshold, will the system go back to the *off* state. For stimulus strengths ranging between the activating and the deactivating thresholds, the response of the system will depend on how the stimulus was varied (Fig. 1B, bistable).

The existence of more than one steady state under particular biological conditions is strongly related to the existence of positive feedback loops, as was shown first for gene regulatory networks^7^ and then demonstrated as a necessary condition^8^. In a positive feedback loop, an increase in the concentration of certain species triggers an effect. This effect eventually feeds back generating a new increase in the concentration of the original species that, in turn, causes an even larger increase. These motifs are common in networks governing cellular behaviors such as proliferation, differentiation, and apoptosis that exhibit all-or-none responses, in which cells commit—usually irreversibly—to one specific fate over a discrete set of possibilities. A common mechanism to generate bistability is by coupling cycles of reversible post-translational modifications^9^. It has been shown that bistability can arise in a dual phosphorylation-dephosphorylation cycle with a non-processive, distributive mechanism for the enzymes. This implies that a MAPK cascade formed by coupling these structures can exhibit bistable behavior, even in the absence of evidently imposed positive feedback loops. The presence of a compartment generates a positive feedback loop resulting in such emergent behavior. From a mathematical point of view and describing the process in the simplest possible way, shuttling in and out of a compartment adds a linear term in the differential equations associated with the system and increases the dimensionality of both the system itself and its parameter space.

Typically, it is difficult to identify the impact that each parameter has in the model’s output for a high-dimensional dynamical system. Different strategies have been proposed, from local approaches like sensitivity analysis^10^ to global ones combining parameter space exploration and statistical tests^11^. More recently, intermediate methods have been developed that employ random sampling to identify multiple parameter sets leading to the desired output and combine it with a global sensitivity estimator^12^.

In this work, we aim to explore the robustness of the emergent bistability due to compartmentalization. After an initial characterization of a compartmentalized phosphorylation cycle by a parameter space exploration, we performed a statistical analysis of the results to determine the sensitivity of the bistability to the different parameters. We also quantified the extent of bistability by computing the area of the hysteresis loop finding that is mainly controlled by shuttling parameters. We additionally unveiled the necessary positive feedback loops using a method that relies on an injectivity criterion. With this information we gradually simplified the model to obtain a reduced system that still displays compartments-induced bistability. In this way, we provide further insight into how spatial organization of cellular systems generates an enriched dynamical response.

## Results

### Phosphorylation-dephosphorylation cycle in two compartments

As a first building block we considered a single phosphorylation-dephosphorylation cycle modeled with Michaelis-Menten reactions, which has been shown to be monostable^3^:

1 $$\begin{array}{c}S+E\mathop{\mathop{\rightleftharpoons }\limits^{{k}_{\text{on,E}}}}\limits_{{k}_{\text{off,E}}}X\mathop{\to }\limits^{{k}_{\text{cat,E}}}{S}^{\ast }+E,\\ {S}^{\ast }+F\mathop{\mathop{\rightleftharpoons }\limits^{{k}_{\text{on,F}}}}\limits_{{k}_{\text{off,F}}}Y\mathop{\to }\limits^{{k}_{\text{cat,F}}}S+F\end{array}$$

These two reactions model the reversible modification of a substrate S into its phosphorylated form S*. Phosphorylation and dephosphorylation are enzymatically catalyzed by E (kinase) and F (phosphatase), respectively, involving the formation of intermediate complexes X, Y. k_on_, k_off_, and k_cat_ are the reaction rate constants for binding, unbinding and catalysis.

**Figure 2:**
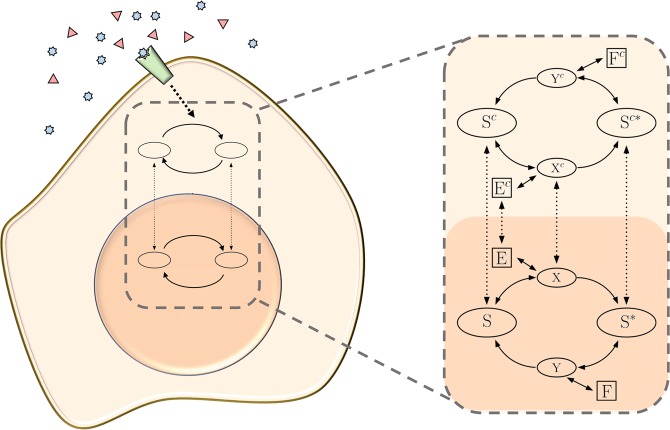
Compartmentalization in eukaryotic cell signaling. (left) Schematic. Receptor proteins located on the cell membrane recognize external signals and transmit them to the interior of the cell. This leads to the activation of different signaling pathways in the cytoplasm, which are in turn responsible for transmitting the signal to the nucleus, where adequate responses are then orchestrated. (right) Compartmentalized reactions. A single phosphorylation/dephosphorylation cycle in two compartments (e.g. cytoplasm and nucleus). In each compartment, the substrate S is phosphorylated by a kinase E via the formation of the intermediate species X and dephosphorylated by a phosphatase F through the accumulation of the intermediate species Y. When intercompartmental shuttle is allowed, the system has been shown to exhibit bistable behavior^4^. Figure adapted from^4^.

We use this building block in two compartments. Eukaryotic cells are characterized by distinct nuclear and cytoplasmic compartments that are separated by a double membrane. This membrane is penetrated by nuclear pore complexes that allow exchange of macromolecules between the two compartments, mediated by specific proteins^13^. In the current work we consider a reversible modification of a substrate, as described above, and allow nucleocytoplasmic shuttling of some species, resulting in a new compartmentalized system with increased dimensionality (Fig. 2). Shuttling is modeled in the simplest possible way, i.e. with linear reactions. This relies on the assumption that the transporters are not rate limiting.

The dynamics of this new system are described by the following ODE system:

$$[\dot{E}]=({k}_{\text{off,E}}+{k}_{\text{cat,E}})[X]-{k}_{\text{on,E}}[E][S]-{k}_{\text{out,E}}[E]+{k}_{\text{in,E}}[{E}^{c}],$$

$$[\dot{X}]=-\,({k}_{\text{off,E}}+{k}_{\text{cat,E}})[X]+{k}_{\text{on,E}}[E][S]-{k}_{\text{out,X}}[X]+{k}_{\text{in,X}}[{X}^{c}],$$

$$[\dot{S}]={k}_{\text{off,E}}[X]-{k}_{\text{on,E}}[E][S]+{k}_{\text{cat,F}}[Y]-{k}_{\text{out,S}}[S]+{k}_{\text{in,S}}[{S}^{c}],$$

$$[{\dot{S}}^{\ast }]={k}_{\text{cat,E}}[X]-{k}_{\text{on,F}}[{S}^{\ast }][F]+{k}_{\text{off,F}}[Y]-{k}_{\text{out,}{S}^{\ast }}[{S}^{\ast }]+{k}_{\text{in,}{S}^{\ast }}[{S}^{c\ast }],$$

$$[\dot{F}]=-{k}_{\text{on,F}}[{S}^{\ast }][F]+{k}_{\text{off,F}}[Y]+{k}_{\text{cat,F}}[Y],$$

$$[\dot{Y}]={k}_{\text{on,F}}[{S}^{\ast }][F]-{k}_{\text{off,F}}[Y]-{k}_{\text{cat,F}}[Y],$$

$$[{\dot{E}}^{c}]=({k}_{\text{off,E}}^{c}+{k}_{\text{cat,E}}^{c})[{X}^{c}]-{k}_{\text{on,E}}^{c}[{E}^{c}][{S}^{c}]+{k}_{\text{out,E}}[E]-{k}_{\text{in,E}}[{E}^{c}],$$

$$[{\dot{X}}^{c}]=-\,({k}_{\text{off,E}}^{c}+{k}_{\text{cat,E}}^{c})[{X}^{c}]+{k}_{\text{on,E}}^{c}[{E}^{c}][{S}^{c}]+{k}_{\text{out,X}}[X]-{k}_{\text{in,X}}[{X}^{c}],$$

$$[{\dot{S}}^{c}]={k}_{\text{off,E}}^{c}[{X}^{c}]-{k}_{\text{on,E}}^{c}[{E}^{c}][{S}^{c}]+{k}_{\text{cat,F}}^{c}[{Y}^{c}]+{k}_{\text{out,S}}[S]-{k}_{\text{in,S}}[{S}^{c}],$$

$$[{\dot{S}}^{c\ast }]={k}_{\text{cat,E}}^{c}[{X}^{c}]-{k}_{\text{on,F}}^{c}[{S}^{c\ast }][{F}^{c}]+{k}_{\text{off,F}}^{c}[{Y}^{c}]+{k}_{\text{out,}{S}^{\ast }}[{S}^{\ast }]-{k}_{\text{in,}{S}^{\ast }}[{S}^{c\ast }],$$

$$[{\dot{F}}^{c}]=\,-{\,k}_{\text{on,F}}^{c}[{S}^{c\ast }][{F}^{c}]+{k}_{\text{off,F}}^{c}[{Y}^{c}]+{k}_{\text{cat,F}}^{c}[{Y}^{c}],$$

2 $$\begin{array}{c}[{\dot{Y}}^{c}]=\,{k}_{\text{on,F}}^{c}[{S}^{c\ast }][{F}^{c}]-{k}_{\text{off,F}}^{c}[{Y}^{c}]-{k}_{\text{cat,F}}^{c}[{Y}^{c}]\end{array}$$

where the species shuttling between compartments are S, S*, E, and X. The superindex “c” indicates that the species are in one of the compartments, the cytosol, and the absence of it indicates that they are in the nucleus. k_in_, and k_out_ are the rate constants for shuttling in and out of a compartment (the one without the “c” label). The ODE system above has been shown to respond in a bistable fashion for a particular choice of parameter values^4^.

**Figure 3:**
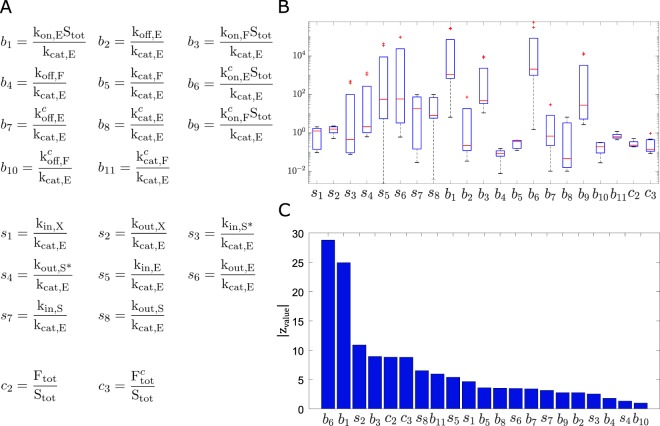
Bistability’s dependence on parameter values. (**A**) Non-dimensional parameters. (**B**) Boxplot obtained from nine sets of parameter values (see Supplementary Table 1) for which the system exhibits bistable responses, obtained by using the CRNToolbox. The boxes delimit the 25^th^ and 75^th^ percentiles, while the red lines represent the medians. The red crosses correspond to outliers, and the dotted lines extend to the more extreme values not considered outliers. This analysis allowed us to restrict the sampling ranges for each parameter. **(C**) Bar plot representing the results of the Mann-Whitney U Test over the bistable fraction. The non-dimensional parameters are sorted by the absolute value of their z-value.

Considering the concentration of the total protein kinase *E*_*tot*_ = [E]+[X]+[E^c^]+[X^c^] as the stimulus, and the concentration of phosphorylated substrate in the nucleus S^*^ as the response, the system involves twenty three control parameters: twelve biochemical reaction rate constants, eight shuttling rates, and three total concentrations of species (the concentrations *S*_*tot*_ = [S]+[S^*^]+[X]+[Y]+[S^c^]+[S^c*^]+[X^c^]+[Y^c^],  *F*_*tot*_ = [F]+[Y],  and ${F}_{tot}^{c}$ = [F^c^]+[Y^c^] of total substrate, total nuclear phosphatase, and total cytoplasmic phosphatase, respectively). The ODE system was converted to a nondimensional form, as detailed in the Supplementary Information. The resulting dimensionless system has twenty-one free parameters and we consider *c*_1_ and s^*^ to be the stimulus and response, respectively. The dimensionless parameters and its definitions are listed in Fig. 3A.

### Characterization of the bistable region

To characterize the bistable region we combined a battery of methods, each one described in detail in the Methods section. Briefly, the Jacobian injectivity criterion allows, if possible, to derive conditions to guarantee monostationarity. CRNToolbox decides whether there exists any combination of rates leading to bistability. MatCont performs continuation analysis. The “going-up and coming-down” analysis is the numerical method to detect bistable input-output curves. LHS is a technique to ensure homogeneous sampling of the parameter space. We combine them as described in the following paragraphs.

First, we characterized the extent of the previously reported bistable behavior^4^ using CRNToolbox and MatCont. CRNToolbox allows to identify parameter sets producing bistability, MatCont allows to obtain the bifurcation diagram starting from that parameter set. We scanned each of the parameters individually, leaving the others unchanged, and generated the respective bifurcation diagrams. We found that bistability is preserved after variations of one or more orders of magnitude for most of the parameters studied individually (see Methods and Supplementary Fig. 1).

While this approach allowed us to assess bistability around a given parameter set, we wanted to evaluate globally the existence of other sets with the same behavior. Due to the high dimensionality of the system, it is computationally difficult to find such sets by randomly varying a combination of parameters in a predetermined range. Therefore, we first employed CRNToolbox to find different bistable points of the system which allowed us to restrict the sampling intervals for each value (see Methods and Supplementary Table 1), as indicated in Fig. 3B. Using those intervals, we studied the behavior of the system using the “going-up and coming-down” analysis with stimulus ranging from 10^−3^ to 10^3^ over roughly 100,000 different randomly selected parameters sets sampled by LHS (details of the “going-up and coming-down” analysis and the LHS sampling method in the Methods section).

To quantify the influence of each parameter, we first binned logarithmically their variation ranges and then calculated the fraction of bistable cases (*f* ^*b*^) for each bin (Supplementary Fig. S2). As expected, the bistable percentage per class turned out to be extremely low (smaller than 8%). Some parameters were found to be critical in controlling the fraction of bistable cases. Using a Mann-Whitney U Test we sorted the parameters according to their influence on the bistability of the system (Fig. 3C, test explained in Methods). Interestingly, shuttling parameters *s*_2_ and *s*_8_ were among the most influential. This was complemented with an enrichment test indicating in which sub-range of each parameter the bistable behavior is enriched (Supplementary Fig. S3, see Methods). While it is certainly possible that some cases labeled as monostable are actually bistable with a stimulus range outside the scanned one, we consider this unlikely as posterior analysis of the resulting bistable cases showed that all switches occur at values of the control parameter well within the scanned range.

**Figure 4:**
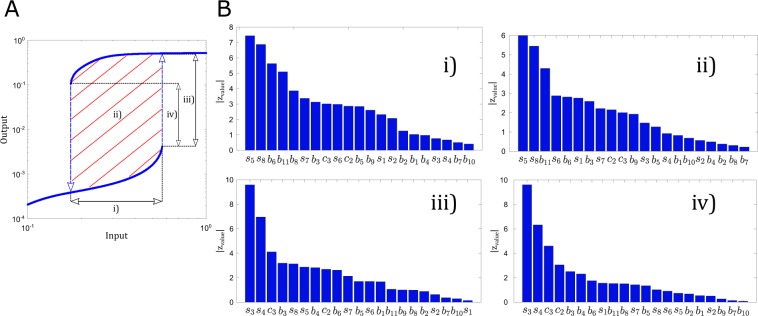
Dependence on the parameters of the different properties of the bistable region. **(A)** Example of bistable curve (blue line), with the properties marked over it: width (i), area of the hysteresis loop (ii, diagonal red lines), the distance between branches at Stim_on_ (iii) and the difference between the output at Stim_off_ and at Stim_on_ (iv). **(B)** Bar plots representing the results of the Mann-Whitney U Test for: the width (i); the area of the hysteresis loop (ii); the distance between branches at Stim_on_ (iii); the difference between the output at Stim_off_ and Stim_on_ (iv). The parameters are sorted by the absolute value of their z-value.

For the parameter sets leading to bistability we also scored the bistable behavior with four different criteria: we computed the input range of bistability (we call it width), the distance between the two bistable branches calculated in two different ways as explained in the Methods section and illustrated in Fig. 4A (we call it height or distance), and the area of the hysteresis loop. A high value of width indicates that there is a non-negligible range of inputs that lead to bistable behavior, a high value of height indicates that the two stable outputs are distinguishable from each other, and a high value of area integrates the information in the other two (width and height). The results of these calculations are in Fig. 4B and Supplementary Fig. S4 and the details of the calculations are explained in the Methods section. Figure 4B contains the influence of each parameter on the mentioned properties (width, height, and area) as sorted by using a Mann-Whitney U Test. Strikingly, the top two parameters controlling the four properties are shuttling parameters. To complement these results, we include the distributions of widths, heights and areas in Supplementary Fig. S4A, and a scatter plot indicating how the top two parameters in Fig. 4B control the corresponding property (Supplementary Fig. S4B).

### Positive feedback loops involved in bistability

**Figure 5:**
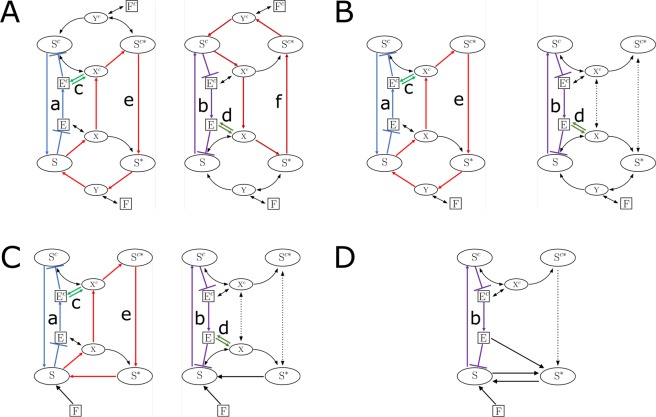
Simplification to a compact model that conserves spatially driven bistability. The original system **(A)** was reduced in four consecutive steps. Left: the feedback **a** is in blue, the feedback **c** in green, the feedback **e** in red. On the right: the feedback **b** in purple, the feedback **d** in dark green, the feedback **f** in dark red. **(B)** Cytoplasmic phosphatase was removed. **(C)** The two-step enzymatic dephosphorylation in the nucleus was replaced by a single step reaction. **(D)** Phosphorylation in the nucleus was replaced by a single step reaction. Finally, the reversible translocation reactions of the phosphorylated and unphosphorylated substrates were replaced by irreversible mechanisms (also in panel **D**). In each case, the relevant feedback loops are indicated with different colors and labeled as a, b, c, d, e, f.

Using the injectivity criterion (see Methods) we identified six positive feedback loops necessary for bistability, which are indicated in Fig. 5A with six different colors over the scheme representing the system (the scheme is duplicated to show only three loops at a time for clarity purposes). Four of them involve shuttling of species between both compartments, confirming that compartmentalization and translocation are responsible for the appearance of bistability in an otherwise monostable system. For example, as the amount of dephosphorylated substrate in the nucleus (*S*) increases, the formation of intermediate complex X also increases, causing the amount of free enzyme in the nucleus to decrease. Such decline entails a decrease in the amount of free enzyme in the cytoplasm (*E*^*C*^), causing the substrate to accumulate there by limiting the formation of the intermediate complex *X*^*C*^. Finally, as the substrate is allowed to shuttle between both compartments, the enlarged concentration of substrate in the cytoplasm causes the concentration of nuclear substrate to increase, closing the positive feedback loop.

To further understand the source of bistability we set out to simplify this model by finding a more compact one that conserves spatially driven bistability. We did so by removing reactions and components instead of reducing the initial model by means of relations between the involved parameter values. The reason for doing so is that the methods we combined only work with reactions instead of differential equations. First, we removed the phosphatase in the cytoplasm. The injectivity criterion identifies in this simplified system five different feedback loops that were also present in the original system (a, b, c, d, and e), three of them (a, b, and e) involving translocation (Fig. 5B and Supplementary Table 2). The remaining loop, f, is broken.

We then further simplified the model by considering the dephosphorylation in the nucleus as a single step reaction:

3 $$\begin{array}{c}F+{S}^{\ast }\mathop{\to }\limits^{{k}_{dephos}}F+S\end{array}$$

The system remained bistable and most of the positive feedback loops were maintained without changes (a, b, c, and d); except for feedback (e) mediated by the phosphatase-substrate Y complex which includes translocation (Fig. 5C and Supplementary Table 2).

As a next step, we removed the intermediate state in the nuclear phosphorylation:

4 $$\begin{array}{c}E+S\mathop{\to }\limits^{{k}_{phos}}E+{S}^{\ast }\end{array}$$

Such simplification kept the bistability but changed the necessary feedback loops (Fig. 5D and Supplementary Table 2). By eliminating the intermediate complex, three cycles (a and e, which involve translocation, and d that does not) were broken. In particular, cycle a is broken because the nuclear enzyme is no longer being sequestered by the substrate to form the intermediate complex X. Cycle b remains closed but is modified, as now the positive feedback loop is completed through the simplified reaction r_phos_. Finally, even though cycle c is still existent, it is no longer relevant for bistability.

Finally, to reduce even more the number of parameters of the system, we replaced the translocation reactions of the phosphorylated and unphosphorylated substrates by the irreversible reactions

5 $$\begin{array}{c}{S}^{c}\mathop{\leftarrow }\limits_{{k}_{\text{out,S}}}S\end{array}$$

6 $$\begin{array}{c}{S}^{c\ast }\mathop{\to }\limits^{{k}_{\text{in,}{S}^{\ast }}}{S}^{\ast }\end{array}$$

We verified that bistability is conserved, and the relevant positive feedback loop is the one from the previous case. This minimal model contains 5 independent variables and 8 nondimensional parameters, while the initial model contains 8 independent variables and 21 nondimensional parameters.

The complete set of reactions for the final system is depicted in Fig. 5D and Supplementary Table 2 and is described by the nondimensional ODE system presented in the Supplementary Information. For this reduced system we used the “going-up and coming-down” analysis with stimulus values ranging from 10^−6^ to 10^1^ over roughly 30,000 different parameter sets sampled by LHS, with all the parameters randomly varied over the range [10^−3^,10^3^]. Following the same approach as before, we calculated the fraction of bistable cases per logarithmically spaced bin for each parameter. The obtained plots are presented in Supplementary Fig. S5. The fraction of bistable cases is still low, with its maxima occurring for high values of b_2_ (8.5%), high values of b_3_ (4.9%), and low values of s_1_ (5.1%). Bistability can be overturned by controlling any of the four biochemical rates: on the one hand, our analysis did not find any bistable cases for b_2_ < 10 or b_3_ < 10^−1^. This is in line with the fact that setting any of these parameters to zero would break the relevant feedback loop (Supplementary Fig. S5). On the other hand, bistability disappears for b_1_ > 10^2^ or b_4_ > 10^2^. In the parameter ranges sampled, s_1_ is the only shuttling parameter with the power to overturn bistability. However, by applying the Jacobian injectivity criterion^14^(see Methods) to this reduced system, we obtain that for extremely low values of s_3,_ the system is necessarily monostationary. The criterion also confirms that multistationarity cannot happen for extremely low values of *b*_2_, *b*_3_, or *s*_1_. Conducting an enrichment test (Supplementary Fig. S5B) further confirmed the results obtained for the fraction of bistable cases per class. Finally, a Mann-Whitney U test selected b_2_, b_3_, s_1_, and b_1_ as the leading parameters in the control of bistability in the ranges considered for the analysis (Supplementary Fig. S5C).

## Discussion

In this work we have explored the extent and origin of compartmentalization-induced bistability. Starting from a previously described system which is composed of two monostable modules acting on different cellular compartments and sharing species through shuttling reactions, we explored its vast parameter space in order to assess the robustness of the emergent bistability. For doing so we developed a method that is a combination of known techniques. The method strongly relies on finding several parameters sets leading to bistable behavior and, with those sets, constructing a restricted parameter space for the analysis. To find several sets leading to bistability we applied the tool CRNT several times with the reactions in different order. As simple as it sounds, this approach resulted to be novel and useful, and allows a parameter space exploration that serves two purposes. First, it gives the opportunity to evaluate robustness of the behavior of interest (bistability in this case), robustness understood as frequency of occurrence in the parameter space. Second, it informs on which parameters, and in which ranges, control the emergence and the characteristics of that behavior.

We found that a very small fraction of the sampled (restricted) parameter space produced a bistable response indicating that the system needs to be properly tuned for this behavior to arise. With properly tuned we mean that the parameters have to take values in very specific and restricted ranges in order to produce bistability, and even so, in that restricted parameter space the bistable cases are lower than 8%. Most importantly, shuttling parameters were among the most influential ones to control this property. Summarizing, we found several sets leading to bistability, and each of them was robust according to a local analysis. The (restricted) parameter space constructed with all of them resulted in low bistability outcomes. What we conclude from this is that the analyzed system possesses “bistability islands”. If it is in one of them, the bistable behavior is robust under parameter fluctuations.

We scored the extent of bistability by quantifying the range of stimulus that produces a bistable response, the distance between the two responses in the bistable regime, and the area of the hysteresis loop. We found that the shuttling parameters control all these properties. This result highlights not only the importance of shuttling to find bistable behavior, but also the fact that, once within the restricted ranges, tuning certain shuttling parameters greatly influences the properties of the bistable region. This output allows the optimization of the bistable properties (for example, if a wide range of stimulus or a sufficiently great distance between branches is required) and provides details into potential hierarchies between parameters in systems with compartmentalization-induced bistability.

To further describe the origin of bistability we proceeded as follows. From the original system with two enzymatic reactions in each compartment and shuttling of certain species between them, totalizing 8 dimensionless variables, 21 dimensionless parameters and 6 relevant feedback loops, we performed gradual simplifications monitoring not to lose bistability and identifying the relevant positive feedback loops responsible for bistability^15^. First, we removed the phosphatase reactions when possible. Secondly, we simplified the enzymatic reactions omitting the intermediate complex formation, which was only possible in the nuclear compartment. Finally, we made unidirectional those shuttling reactions that do not cut the feedback loop. Altogether, the simplified system again connects two monostable systems with minimal nonlinearities by a reduced set of linear shuttling reactions that circulates all the species around the two compartments.

The procedure used to obtain a reduced system, while not comprehensive, combines biological knowledge about the involved reactions with the outcome of the feedbacks analysis. In this way, we were able to bypass the current limitations of the Chemical Reaction Network Toolbox (CRNT), meaning that this method only works with reactions instead of ODEs, and the complexity of the model which obstructs even a quasi-steady state approximation. This regular approximation in an enzymatic reaction is complicated in the equations we studied without further (non-rigorous) assumptions, because the shuttling reactions couple all the enzymatic reactions in the model.

The procedure we followed, instead, was to remove reactions or components, for example a phosphatase (this could represent a real system in which the phosphatase is found exclusively in one particular compartment, like is the case of Hog1’s phosphatase Ptp2, which is known to be nuclear^16^), one at a time, monitoring not to lose bistability. We tried several combinations, and the ones we report are those that ensure bistability. It is not demonstrated that the model is minimal, but we could not remove any extra variable or reaction without losing bistability completely, so, in a sense it is minimal. The detailed model has six relevant feedback loops, the reduced model, only one. This reduced model is bistable based on the same mechanisms that ensure bistability in the more detailed one: enzymatic (and simplified enzymatic) reactions that transform variables and shuttling reactions that circulate them, generating in this way a positive feedback loop that is the origin of bistability.

Summarizing, the novelty of this work relies on three aspects. First, the approach itself. In the article by Feliu and co-workers they used CRNT to find a parameter set leading to bistability followed by local analysis around those parameter values. It is explained that a high-dimensional parameter space exploration is likely not successful. In this article, we could perform a parameter space exploration by first defining a reduced parameter space obtained by successive applications of CRNT. Second, with this approach, an analysis that is not completely global but is not local, was performed. It provided an insight on how robust bistability is in the system considered, and on how the different types of parameters, those associated with reactions, with shuttling and with concentrations, control not only the emergence of bistability but also its properties. Third, a very compact model with compartment-induced bistability was derived.

Bistable systems are one of the main building blocks of more complex behaviors such as oscillations, memory, and digitalization. Therefore, we expect that the proposed methodology and the results arising from it provide insight into how these behaviors can arise from compartmentalization.

## Methods

### Chemical reaction network toolbox

The Chemical Reaction Network Theory (CRNT) developed by Horn, Jackson, and M. Feinberg’s group draws relationships between the structure of chemical reaction networks and their qualitative properties, such as the existence, uniqueness, multiplicity, and stability of their fixed points. Among other questions, this theory allows us to decide whether there exists any combination of rate constant values such that a certain reaction network has the capacity for bistability. CRNT has been implemented by the authors as a computational Toolbox. In this work we use the Chemical Reaction Network Toolbox v2.3. The CRNToolbox produces distinct solutions depending on the order in which the reactions are fed. For this system, nine different solutions were found (see Supplementary Table 1).

### Going-up and coming-down analysis

To identify bistable behaviors and their thresholds (see below) in those structures capable of bistability as proposed by CRNToolbox, we implemented the “going-up and coming-down” analysis^17^ in MATLAB (version 2015a, The MathWorks, Inc., Natick, Massachusetts, United States). Briefly, the steady-state response curve upon stimulus increase (“going-up” branch) is compared with the one obtained when the stimulus is decreased (“coming-down” branch). Importantly, for each stimulus, the initial condition is the steady-state output obtained with the previous stimulation. If the system is monostable both branches will coincide, as in this case the steady state solution is unique for each stimulus. In contrast, for a toggle switch the branches will not match for a certain stimulus range. At the lower (*Stim*_*off*_) and upper (*Stim*_*on*_) limits of such range, the “coming-down” and “going-up” branches will respectively show a discontinuity.

We implemented the analysis by describing the biological model of interest as a system of coupled ordinary differential equations (ODE) defining the time evolution of the concentration of each biochemical species. The dynamical behavior is not only determined by the structure of the ODE system but also by its parameters (e.g. rate constants, total concentrations). Among those, one is considered as the stimulus while the others are the parameter sets at which the capability of a bistable response is assessed.

At its core, the computational code we developed evolves numerically the ODE system for a given parameter set and a predefined stimulus value until the relative change in concentrations is negligible and thereby a steady-state is considered to be found. The process is repeated first ascending and then descending through a predefined stimuli vector. For the first stimulus value, the initial concentrations are set so that all enzymes are in the cytoplasmic inactive state. A small perturbation from the resulting response steady state is then used as the initial concentration for the next stimulus value and evolved to a new steady state. Discontinuities in both branches of the stimulus-response curve are identified as those values that deviate significantly from the trend given by neighboring values. Specifically, a discontinuity is said to exist at the stimulus value Stim_i_ if the difference between the response for Stim_i_ and Stim_i−1_ is at least five times larger than the difference between the output for Stim_i−1_ and Stim_i−2_, and is higher than 10^−3^. This last threshold has been selected empirically to reduce the presence of false positives. If one discontinuity is found in each of the branches, the system is labeled bistable. In contrast if no discontinuities are found, the parameter set is labeled monostable. If only one is found, the system is flagged for further analysis to evaluate if the system is actually a one-way-switch or the original stimulus vector was not appropriate (i.e. the range being considered does not include one discontinuity or the components of the vector are too separated). We restricted the analysis to responses that were greater or equal to 10^−2^ for at least one stimulus value.

### Parameter sampling

Using Latin Hypercube Sampling (LHS)^11,18^, to ensure an homogenous sampling, we scanned the multidimensional parameter space and identified regions of bistability. To investigate the existence of patterns, we performed an enrichment test for each parameter^19^ followed by a test of the influence of each parameter.

#### Enrichment test

Given a set of values of a parameter, enrichment tests^19^ can be used to assess whether the members of a subset of the original set are randomly distributed or whether they are clustered in specific regions. Here, we performed enrichment tests to investigate the existence of patterns in the parameter values that give rise to bistable behavior. Sampled values were separated into logarithmically spaced bins (classes). Let N be the total number of values sampled for a parameter, and M the number of those for which the system was found to be bistable. Also, let *y*_*i*_ and *x*_*i*_ be the number of those values that correspond to the i-th class in N and M, respectively, so that $\sum _{i}{y}_{i}=N$ and $\sum _{i}{x}_{i}=M$. The main idea is to determine whether the behavior in each class is enriched with respect to the case in which M values of the parameter under analysis are selected independently and uniformly in a random way without replacement among the N values sampled (null hypothesis). If that was the case, the probability of observing at least *x*_*i*_ values in the i-th class giving place to bistability would follow a hypergeometric distribution:

7 $$\begin{array}{c}P(n={x}_{i}|{y}_{i},\,M,\,N)=\frac{(\begin{array}{c}{y}_{i}\\ {x}_{i}\end{array})(\begin{array}{c}N-{y}_{i}\\ M-{x}_{i}\end{array})}{(\begin{array}{c}N\\ M\end{array})}.\end{array}$$

We can then calculate a p-value *p*_*i*_ to measure the statistical significance of the probability of observing at least *x*_*i*_ values giving place to bistability in the i-th class, when the null hypothesis is assumed as the real underlying distribution. In this work, we considered the i-th classed to be enriched if *p*_*i*_ < 10^−4^.

#### Test of the influence of each parameter in controlling bistability

To assess the influence of each parameter in the control of bistability, we partitioned the distribution of values for each parameter in two groups: the positive group contained the values of the parameter for which the system behaved in a bistable fashion, while values for which the system was monostable were included in the negative group. Statistical significance was tested using a Mann-Whitney U^20^ test implemented in MATLAB (Statistical Toolbox, version 2015a, The MathWorks, Inc., Natick, Massachusetts, United States).

### MatCont

We used MatCont, a graphical MATLAB software package implemented for the interactive numerical study of dynamical systems. Briefly, the algorithm finds the steady state and then performs a continuation analysis while changing the parameter of choice to build a bifurcation diagram.

### Methods for the width, area and distance analysis

After removing the parameter sets with responses lower than 10^−2^, we studied the bistable outputs. For each parameter set, we measured the following four properties: the width of the bistable region (Stim_on_-Stim_off_), the area of the hysteresis loop (area of the “coming-down” branch - area of the “going up branch”), and the distance between the two branches (distance_1_ = difference between branches at Stim_on_; distance_2_ = difference between the output at Stim_off_ and at Stim_on_).

First, we plotted the distribution of values for the corresponding variable (width, area, distance) in logarithmic classes. This gave us 7 classes for the width (between 10^−6^ and 10^1^), 9 for the area (between 10^−8^ and 10^1^), 6 for distance_1_ (between 10^−3^ and 10°, divided in halves: 10^−3^ – 0.5 × 10^−2^, 0.5 × 10^−2^ – 10^−2^, etc.) and 7 for distance_2_ (between 10^−4^ and 10°, also divided in halves, with an empty 10^−4^ – 0.5 × 10^−3^ class).

Then, we defined two groups among the parameter sets: one containing the two classes with the higher values for each variable, the other containing the rest of the classes. The value separating the two groups for all four variables is the same: 10^−1^. Using a Mann-Whitney U test implemented in MATLAB, we could analyze the influence of each parameter in the values of the four variables.

Finally, for the two parameters with the highest absolute z_value_, we plotted one parameter versus the other in a colormap as a function of the corresponding variable. This allowed us to interpret the kind of control that the two parameters have over the variables.

Some considerations: for the first step in this method, the classes for the distances were divided in halves to have a higher number of classes; in the third step, only the two parameters with the highest absolute z_values_ showed a pattern in the colormap. Also, out of the 1117 parameter sets with bistable curves (with an S shape), 78 showed negative values for the area, due to small values in the width, which resulted in integration errors. These cases were not considered.

### Jacobian injectivity criterion

Given a network of interacting species, the Jacobian injectivity criterion^14^ allows us to either preclude multistationarity or derive conditions on the rate constants that guarantee monostationarity^4^. Given $f=({f}_{1},\ldots ,\,{f}_{n}\,):{{\mathbb{R}}}^{n}\to {{\mathbb{R}}}^{n}$ a differentiable function, the Jacobian at a point $x=({x}_{i},\ldots ,\,{x}_{n})$ in ${{\mathbb{R}}}^{n}$, ${J}_{x}(f)$, is defined as the $n\times n$ matrix having $\frac{\partial {f}_{i}}{\partial {x}_{j}}$ as the (*i*,*j*) element. If *f* is a polynomial function, then all elements of ${J}_{x}(f)$ are polynomials in *x*, and so is its determinant. The Jacobian injectivity criterion states that if all ${f}_{i}$ are polynomials of order less than or equal to two, and the determinant of the Jacobian is not zero in a convex domain $\Omega \subseteq {{\mathbb{R}}}^{n}$, then *f* is injective in $\Omega$.

The positive steady states for a system of biochemical reactions described by the law of mass action are given by the positive solutions of a system of polynomial equations ${f}_{\kappa ,i}(x)=0,\,i=1,\ldots ,n,$ where *n* is the number of species in the system. The coefficients of polynomials ${f}_{\kappa ,i}$ depend on the form of the conservation laws and on the rate constants $\kappa =\{{k}_{j}\}$, where *k*_*j*_ is the rate constant for reaction *r*_*j*_. If the function ${f}_{\kappa }=({f}_{\kappa ,1},\ldots {f}_{\kappa ,n}\,)$ is injective over the positive real numbers ${{\mathbb{R}}}_{+}^{n}$, then there cannot be more than one positive solution to the equation ${f}_{\kappa }(x)=(0,\ldots ,0),$ and multistationarity cannot happen. Hence, the Jacobian injectivity criterion allows us to find conditions that assure the injectivity of *f*_*k*_, and consequently the monostationarity of the network. If all the coefficients of the determinant of ${J}_{x}({f}_{\kappa })$ have the same sign, it will not vanish for any positive value of *x*, and therefore, *f*_*k*_ will be injective in ${{\mathbb{R}}}_{+}^{n}$ and the system will not present more than one steady state. However, if that is not the case, the criterion will still allow us to find conditions for the rate constants that are necessary for multistationarity to occur^4^.

It is possible to use this criterion to create an algorithm that selects the structural positive feedback loops that are relevant for the emergence of bistability. Structural because they do not depend on the exact functional form of the flows, and relevant because, if they are all broken, bistability is lost. For this work, we used a Maple implementation of this algorithm^15^.

## Supplementary information

Supplementary Information.
