# Supplementary material for: Robustness in spatially driven bistability in signaling systems
Source: Sci Rep. 2020 Mar 27;10:5591. doi: 10.1038/s41598-020-62412-1 (PMC7101377; doi:10.1038/s41598-020-62412-1)
Supplement: Supplementary file 1 — Supplementary Information. [file 41598_2020_62412_MOESM1_ESM.docx]

**Supplementary Information**

**Robustness in spatially driven bistability in signaling systems**

Debora Tenenbaum, Juan Ignacio Marrone, Hernán E. Grecco, Alejandra C. Ventura

Supplementary Text 1

*Non-dimensional ODE system for a phosphorylation-dephosphorylation cycle in two compartments with intercompartmental shuttling.*

$$\frac{de}{d\tau}=\left( b_{2}+1 \right)x-b_{1}es-s_{6}e+s_{5}e^{c},$$

$$\frac{dx}{d\tau}= -\left( b_{2}+1 \right)x+b_{1}es-s_{2}x+s_{1}x^{c},$$

$$\frac{ds}{d\tau}=b_{2}c_{1}x-b_{1}c_{1}es+b_{5}c_{2}y-s_{8}s+s_{7}s^{c},$$

$$\frac{ds^{*}}{d\tau}=c_{1}x-b_{3}c_{2}fs^{*}+b_{4}c_{2}y-s_{4}s^{*}+s_{3}s^{c*},$$

$$\frac{df}{d\tau}= -b_{3}fs^{*}+\left( b_{4}+b_{5} \right)y,$$

$$\frac{dy}{d\tau}=b_{3}fs^{*}-\left( b_{4}+b_{5} \right)y,$$

$$\frac{de^{c}}{d\tau}=\left( b_{7}+b_{8} \right)x^{c}-b_{6}e^{c}s^{c}+s_{6}e-s_{5}e^{c},$$

$$\frac{dx^{c}}{d\tau}= -\left( b_{7}+b_{8} \right)x^{c}+b_{6}e^{c}s^{c}+s_{2}x-s_{1}x^{c},$$

$$\frac{ds^{c}}{d\tau}=b_{7}c_{1}x^{c}-b_{6}c_{1}e^{c}s^{c}+b_{11}c_{3}y^{c}+s_{8}s-s_{7}s^{c},$$

$$\frac{ds^{c*}}{d\tau}=b_{8}c_{1}x^{c}-b_{9}c_{3}f^{c}s^{c*}+b_{10}c_{3}y^{c}+s_{4}s^{*}-s_{3}s^{c*},$$

$$\frac{df^{c}}{d\tau}= -b_{9}f^{c}s^{c*}+\left( b_{10}+b_{11} \right)y^{c},$$

$$\begin{aligned} \frac{dy^{c}}{d\tau}=b_{9}f^{c}s^{c*}-\left( b_{10}+b_{11} \right)y^{c} \#\left( 3 \right) \end{aligned}$$

where $\tau=k_{cat,E}t$ and we have defined the nondimensional concentrations as:

$$e=\frac{\left[ E \right]}{E_{tot}}, e^{c}=\frac{\left[ E^{c} \right]}{E_{tot}}, x=\frac{\left[ X \right]}{E_{tot}}, x^{c}=\frac{\left[ X^{c} \right]}{E_{tot}},$$

$$f=\frac{\left[ F \right]}{F_{tot}}, f^{c}=\frac{\left[ F^{c} \right]}{F_{tot}^{c}}, y=\frac{\left[ Y \right]}{F_{tot}}, y^{c}=\frac{\left[ Y^{c} \right]}{F_{tot}^{c}} ,$$

$$\begin{aligned} s=\frac{\left[ S \right]}{S_{tot}}, s^{c}=\frac{\left[ S^{c} \right]}{S_{tot}}, s^{*}=\frac{\left[ S^{*} \right]}{S_{tot}}, s^{c*}=\frac{\left[ S^{c*} \right]}{S_{tot}}, \#\left( 4 \right) \end{aligned}$$

the nondimensional rate constant as:

$$b_{1}=\frac{k_{on,E}S_{tot}}{k_{cat,E}}, b_{2}=\frac{k_{off,E}}{k_{cat,E}}, b_{3}=\frac{k_{on,F}S_{tot}}{k_{cat,E}}, b_{4}=\frac{k_{off,F}}{k_{cat,E}},$$

$$b_{5}=\frac{k_{cat,F}}{k_{cat,E}}, b_{6}=\frac{k_{on,E}^{c}S_{tot}}{k_{cat,E}}, b_{7}=\frac{k_{off,E}^{c}}{k_{cat,E}}, b_{8}=\frac{k_{cat,E}^{c}}{k_{cat,E}},$$

$$\begin{aligned} b_{9}=\frac{k_{on,F}^{c}S_{tot}}{k_{cat,E}}, b_{10}=\frac{k_{off,F}^{c}}{k_{cat,E}}, b_{11}=\frac{k_{cat,F}^{c}}{k_{cat,E}}, \#\left( 5 \right) \end{aligned}$$

the nondimensional shuttling rates as:

$$s_{1}=\frac{k_{in,X}}{k_{cat,E}}, s_{2}=\frac{k_{out,X}}{k_{cat,E}}, s_{3}=\frac{k_{in,S^{*}}}{k_{cat,E}}, s_{4}=\frac{k_{out,S^{*}}}{k_{cat,E}},$$

$$\begin{aligned} {s_{5}=\frac{k_{in,E}}{k_{cat,E}}, s}_{6}=\frac{k_{out,E}}{k_{cat,E}}, s_{7}=\frac{k_{in,S}}{k_{cat,E}}, s_{8}=\frac{k_{out,S}}{k_{cat,E}}, \#\left( 6 \right) \end{aligned}$$

and finally, the relative total concentrations as:

$$\begin{aligned} c_{1}=\frac{E_{tot}}{S_{tot}}, c_{2}=\frac{F_{tot}}{S_{tot}}, c_{3}=\frac{F_{tot}^{c}}{S_{tot}}. \#(7) \end{aligned}$$

The conservation equations become:

$$e+x+e^{c}+x^{c}=1,$$

$$f+y=1,$$

$$f^{c}+y^{c}=1,$$

$$\begin{aligned} s+s^{*}+s^{c}+s^{c*}+(x+x^{c})\frac{E_{tot}}{S_{tot}}+y\frac{F_{tot}}{S_{tot}}+y^{c}\frac{F_{tot}^{c}}{S_{tot}}=1, \#\left( 8 \right) \end{aligned}$$

Supplementary Text 2

*Non-dimensional ODE system for more compact model that conserves spatially driven bistability*

$$\frac{de^{c}}{d\tau}=\left( b_{1}+1 \right)x^{c}-b_{2}e^{c}s^{c}-s_{3}e^{c}+s_{4}e,$$

$$\frac{ds^{c}}{d\tau}=b_{1}c_{1}x^{c}-b_{2}c_{1}e^{c}s^{c}+s_{1}s,$$

$$\frac{dx^{c}}{d\tau}= -\left( b_{1}+1 \right)x^{c}+b_{2}e^{c}s^{c},$$

$$\frac{ds^{c*}}{d\tau}=c_{1}x^{c}-s_{2}s^{c*},$$

$$\frac{ds}{d\tau}= -b_{3}se+b_{4}s^{*}-s_{1}s,$$

$$\frac{de}{d\tau}=s_{3}e^{c}-s_{4}e,$$

$$\begin{aligned} \frac{ds^{*}}{d\tau}=b_{3}se-b_{4}s^{*}+s_{2}s^{c*}, \#\left( 13 \right) \end{aligned}$$

where $\tau=k_{cat,E}^{c}t$ and we have defined the nondimensional concentrations as:

$$e=\frac{\left[ E \right]}{E_{tot}}, e^{c}=\frac{\left[ E^{c} \right]}{E_{tot}}, x^{c}=\frac{\left[ X^{c} \right]}{E_{tot}}, f=\frac{\left[ F \right]}{F_{tot}}, f^{c}=\frac{\left[ F^{c} \right]}{F_{tot}^{c}},$$

$$\begin{aligned} s=\frac{\left[ S \right]}{S_{tot}}, s^{c}=\frac{\left[ S^{c} \right]}{S_{tot}}, s^{*}=\frac{\left[ S^{*} \right]}{S_{tot}}, s^{c*}=\frac{\left[ S^{c*} \right]}{S_{tot}}, \#(14) \end{aligned}$$

the nondimensional rate constants as:

$$\begin{aligned} b_{1}=\frac{k_{off,E}^{c}}{k_{cat,E}^{c}}, b_{2}=\frac{k_{on,E}^{c}S_{tot}}{k_{cat,E}^{c}}, b_{3}=\frac{k_{phos}E_{tot}}{k_{cat,E}^{c}}, b_{4}=\frac{k_{dephos}F_{tot}}{k_{cat,E}^{c}}, \#\left( 15 \right) \end{aligned}$$

the nondimensional shuttling rates as:

$$\begin{aligned} s_{1}=\frac{k_{out,S}}{k_{cat,E}^{c}}, s_{2}=\frac{k_{in,S^{*}}}{k_{cat,E}^{c}}, s_{3}=\frac{k_{in,E}}{k_{cat,E}^{c}}, s_{4}=\frac{k_{out,E}}{k_{cat,E}^{c}}, \#\left( 16 \right) \end{aligned}$$

and the relative concentration as:

$$\begin{aligned} c_{1}=\frac{E_{tot}}{S_{tot}}. \#(17) \end{aligned}$$

Thus, the conservation equations become:

$$e+e^{c}+x^{c}=1,$$

$$\begin{aligned} s+s^{*}+s^{c}+s^{c*}+c_{1}x^{c}=1.\#\left( 18 \right) \end{aligned}$$

Here again we consider $c_{1}$ and $s^{*}$ to be the stimulus and response, respectively.

Supplementary Tables

| Supplementary Table 1 List of parameter sets with bistable solutions | | | | | | | | | |
| --- | --- | --- | --- | --- | --- | --- | --- | --- | --- |
| Param. | Parameter sets | | | | | | | | |
| $\boldsymbol{b}_{\boldsymbol{1}}$ | 680.43 | 281070 | 7113.8 | 1107.8 | 8473.8 | 988.89 | 681.98 | 260580 | 6.5458 |
| $\boldsymbol{b}_{\boldsymbol{2}}$ | 0.3249 | 73.103 | 0.2188 | 0.1193 | 0.2188 | 0.3249 | 0.1222 | 73.103 | 0.0344 |
| $\boldsymbol{b}_{\boldsymbol{3}}$ | 258.43 | 8123.8 | 35.288 | 10.986 | 29.89 | 375.58 | 45.712 | 9295.7 | 47.557 |
| $\boldsymbol{b}_{\boldsymbol{4}}$ | 0.1543 | 0.0835 | 0.0896 | 0.1061 | 0.0637 | 0.1543 | 0.0580 | 0.0835 | 0.0076 |
| $\boldsymbol{b}_{\boldsymbol{5}}$ | 0.4203 | 0.1255 | 0.4093 | 0.3677 | 0.2910 | 0.4203 | 0.3549 | 0.1255 | 0.1679 |
| $\boldsymbol{b}_{\boldsymbol{6}}$ | 1124.4 | 304720 | 9136.9 | 2071.8 | 10884 | 1104 | 646.48 | 571520 | 1.4695 |
| $\boldsymbol{b}_{\boldsymbol{7}}$ | 1.1791 | 0.0102 | 29.146 | 0.2930 | 29.146 | 1.1791 | 0.6835 | 0.0102 | 0.5496 |
| $\boldsymbol{b}_{\boldsymbol{8}}$ | 0.0465 | 0.0102 | 3.3155 | 6.6683 | 3.3155 | 0.0465 | 0.0175 | 0.0102 | 0.3473 |
| $\boldsymbol{b}_{\boldsymbol{9}}$ | 280.02 | 12067 | 2.9970 | 6.0117 | 2.7276 | 274.96 | 26.045 | 13808 | 27.653 |
| $\boldsymbol{b}_{\boldsymbol{10}}$ | 0.3249 | 0.3104 | 0.1014 | 0.0282 | 0.0935 | 0.3249 | 0.1931 | 0.3104 | 0.0840 |
| $\boldsymbol{b}_{\boldsymbol{11}}$ | 0.4401 | 0.8438 | 0.6253 | 0.7791 | 0.5767 | 0.4401 | 0.5926 | 0.8438 | 1.1794 |
| $\boldsymbol{s}_{\boldsymbol{1}}$ | 0.1390 | 1.6072 | 2.0783 | 0.9761 | 2.0783 | 0.1390 | 0.0956 | 1.6072 | 1.2595 |
| $\boldsymbol{s}_{\boldsymbol{2}}$ | 2.0367 | 1.5823 | 2.2798 | 0.5239 | 2.2798 | 2.0367 | 1.1889 | 1.5823 | 0.5344 |
| $\boldsymbol{s}_{\boldsymbol{3}}$ | 3.4041 | 391.83 | 0.0943 | 0.0941 | 0.0781 | 3.4041 | 0.4537 | 485.85 | 0.1565 |
| $\boldsymbol{s}_{\boldsymbol{4}}$ | 2.6916 | 1065.1 | 1.0374 | 2.1180 | 1.0374 | 3.9839 | 1.0987 | 1320.7 | 0.6336 |
| $\boldsymbol{s}_{\boldsymbol{5}}$ | 6.3703 | 34639 | 325.12 | 55.696 | 325.12 | 6.3703 | 2.9486 | 42950 | 0.0023 |
| $\boldsymbol{s}_{\boldsymbol{6}}$ | 3.9165 | 94159 | 334.03 | 58.244 | 334.03 | 3.9165 | 1.4730 | 94593 | 0.6107 |
| $\boldsymbol{s}_{\boldsymbol{7}}$ | 98.275 | 0.0296 | 18.408 | 9.2737 | 18.408 | 98.275 | 66.448 | 0.0486 | 0.1794 |
| $\boldsymbol{s}_{\boldsymbol{8}}$ | 68.447 | 7.3271 | 7.9152 | 1.4499 | 7.9152 | 101.31 | 68.569 | 7.3271 | 0.0038 |
| $\boldsymbol{c}_{\boldsymbol{2}}$ | 0.2007 | 0.1910 | 0.4460 | 0.2770 | 0.5265 | 0.2044 | 0.2304 | 0.2070 | 0.3143 |
| $\boldsymbol{c}_{\boldsymbol{3}}$ | 0.1104 | 0.1396 | 0.4750 | 0.9450 | 0.4323 | 0.1124 | 0.1234 | 0.1513 | 0.0857 |

Eight bistable points of the system found using CRNToolbox (columns 2-9), which allowed us to restrict the sampling intervals for each value. The parameter set of the last column was found in (Harrington et al. 2013).

**Supplementary Table 2**

**
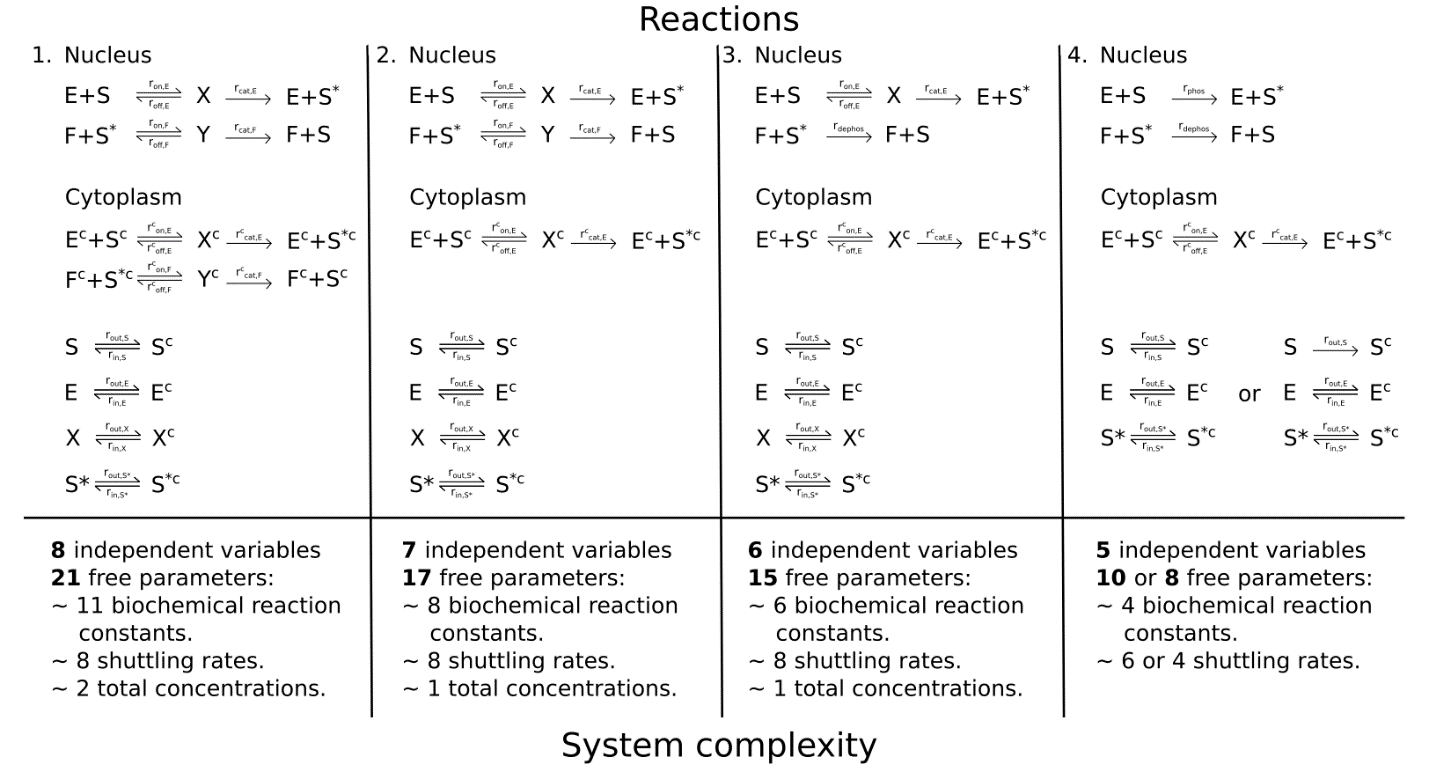
**

The reactions in the original model (first column, first row) were simplified sequentially to obtain a more compact model that conserves spatially driven bistability (fourth column). The simplifications performed in each step are shown, alongside with the resulting numbers of variables and parameters.

Supplementary Figures


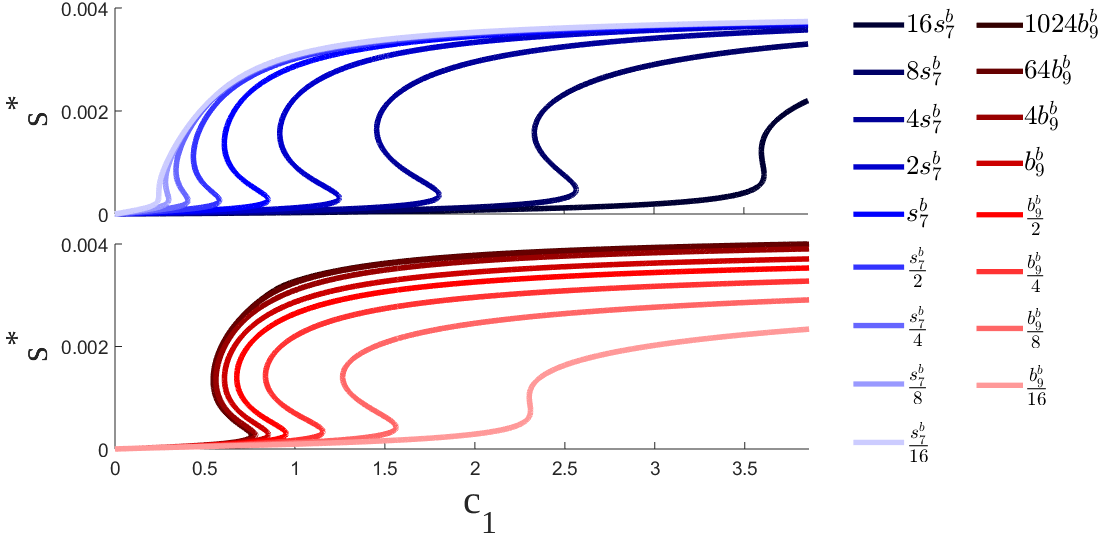


**Figure S1**

**Bifurcation diagrams** generated after varying the non-dimensional parameters s_7_ and b_9_ individually with respect to their original values in (Harrington et al. 2013). Bistability is preserved after variations of one or more orders of magnitude for most of the parameters. Results obtained with Matcont.


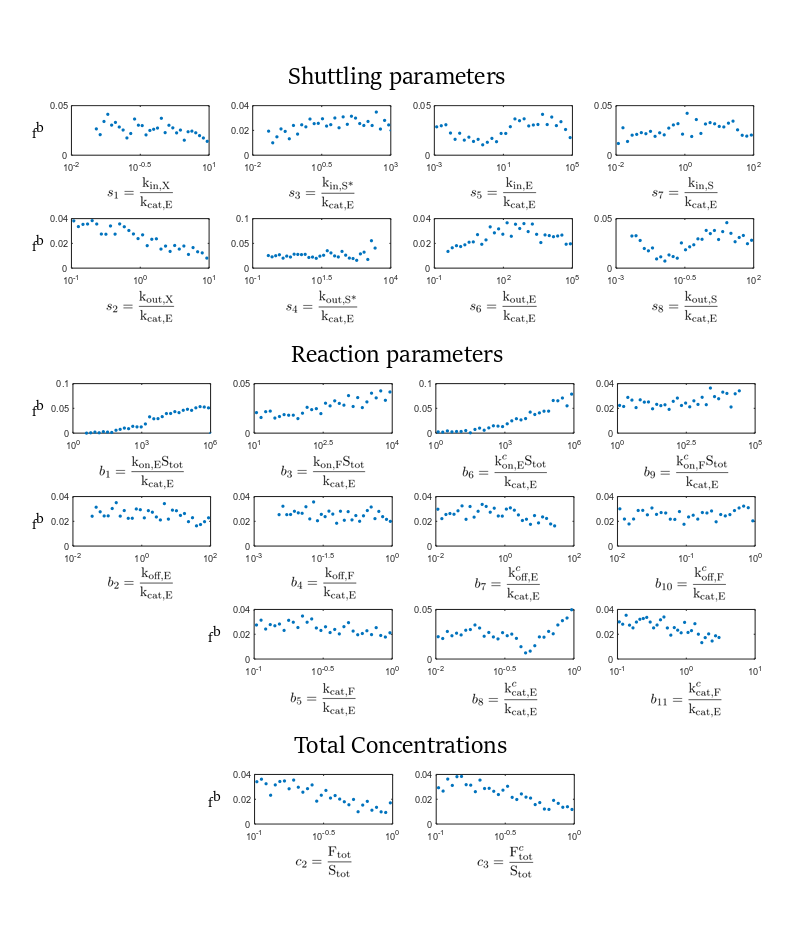
**Figure S2**

**Bistable fraction plots** for individual parameters were constructed as follows. First, the behavior of the system in the dimensionless parameter space was studied by applying the “coming up and going down” method over roughly 100,000 different parameter sets sampled via LHS. Then, the sampling range for each individual parameter was logarithmically binned, and both the total cases in which the parameter adopted a value inside the i^th^ bin (m_i_), and those of them in which the system showed bistable behavior (n_i_) were counted. Finally, the bistable fraction f^b^=n_i_/m_i_ was plotted for each bin.


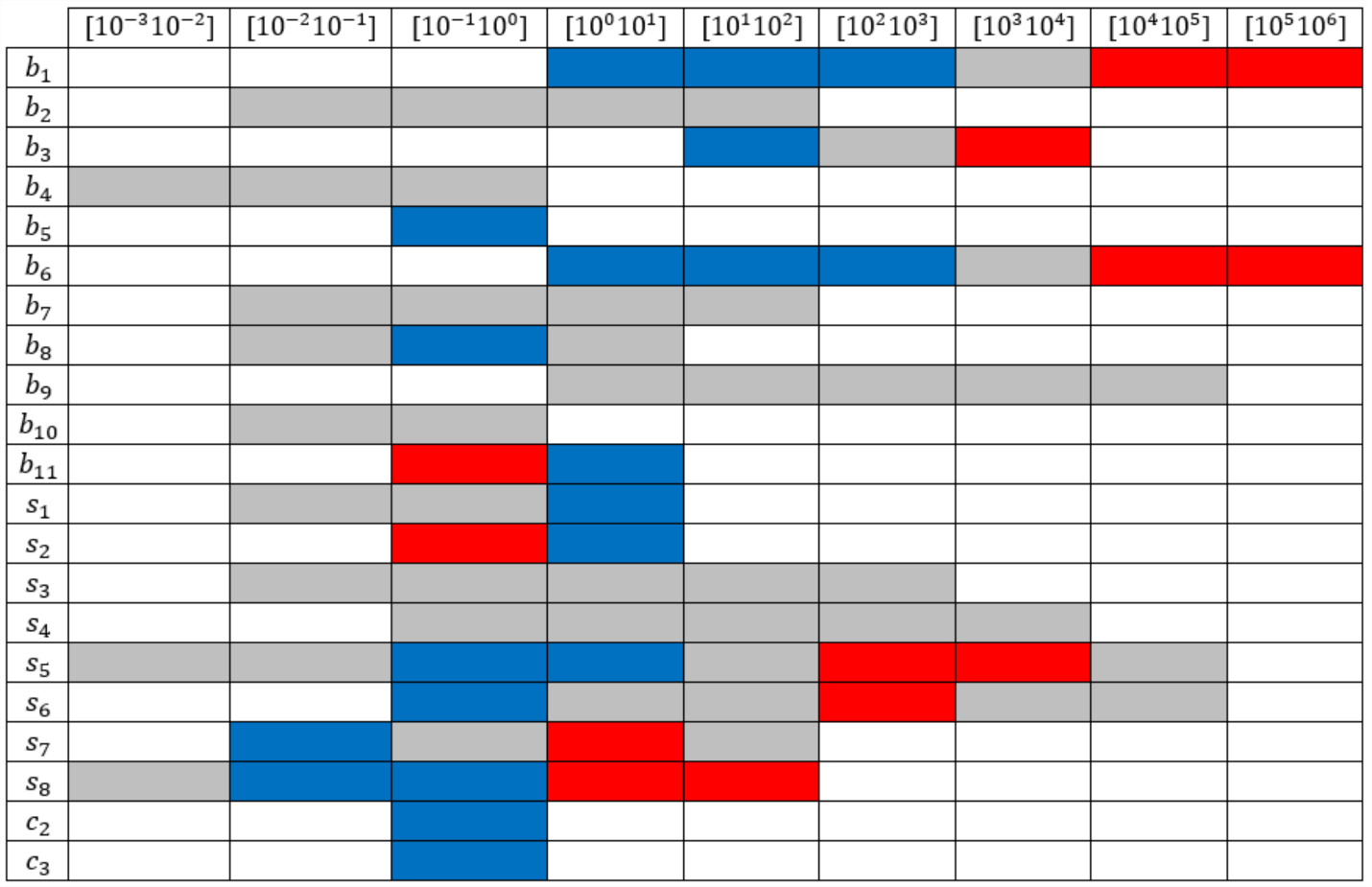


**Figure S3**

Results of the enrichment test for the sampling ranges explored in Fig. 3B. The red boxes represent the enriched classes for each parameter, while the blue ones show the impoverished classes. Grey boxes represent classes that are neither enriched nor impoverished, and white boxes represent empty classes.


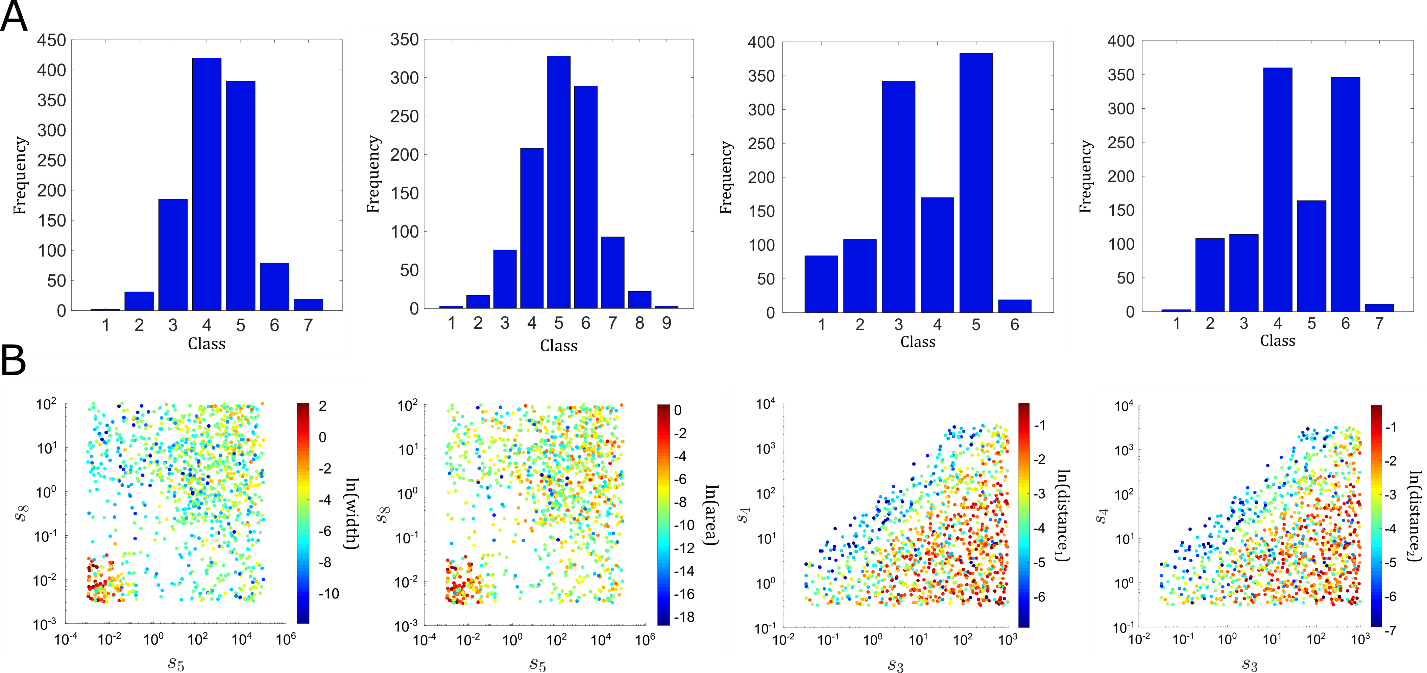


**Figure S4**

**Characterization of the bistable region for the S-type bistable cases**. From left to right, results for: the width; the area of the hysteresis loop; the distance between branches at Stim_on_; the difference between the output at Stim_off_ and Stim_on_. **A**) Distribution of values of each property, in logarithmic classes (as explained in Methods). **B**) Scatter plots for the two parameters with the highest absolute z_value_ (see Fig. 4B), with a colormap for the natural logarithm of the corresponding property.


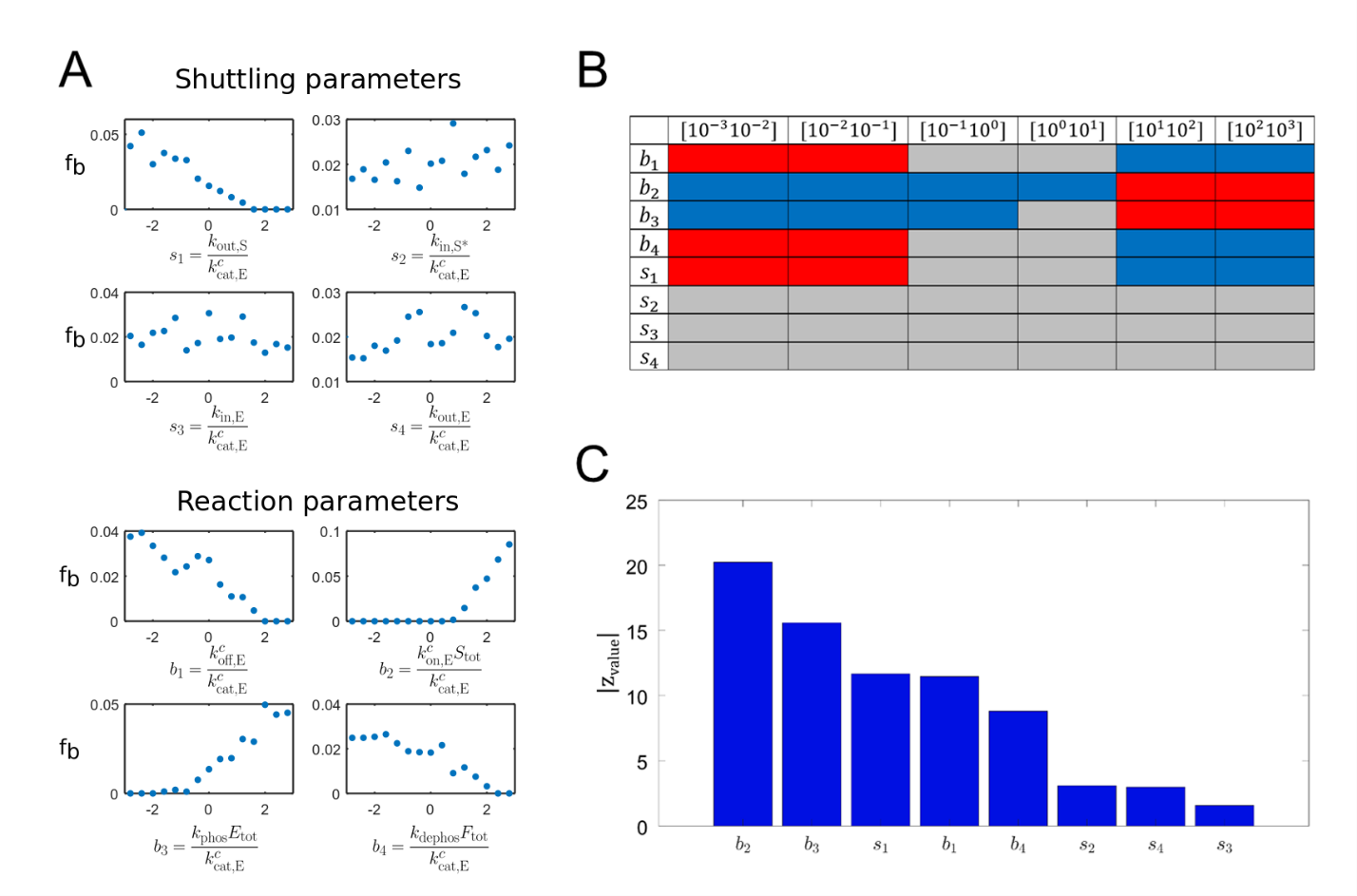


**Figure S5**

**Analysis of the compact system with spatially driven bistability**. **A**) Bistable fraction plots for individual parameters of the compact system. The x-axis in each plot represents the log10 of the non-dimensional parameter values **B**) Results of the enrichment test for the compact system. The red boxes represent the enriched classes for each parameter, while the blue ones show the impoverished classes. Grey boxes represent classes that are neither enriched nor impoverished. **C**) Bar plot representing the results of the Mann-Whitney U Test, for the compact system. The parameters are sorted by the absolute value of their z_value_.
